# The Efficacy of New Chinese Diabetes Risk Score in Screening Undiagnosed Type 2 Diabetes and Prediabetes: A Community-Based Cross-Sectional Study in Eastern China

**DOI:** 10.1155/2020/7463082

**Published:** 2020-04-29

**Authors:** Tao Mao, Jiayan Chen, Haijian Guo, Chen Qu, Chu He, Xuepeng Xu, Guoping Yang, Shiqi Zhen, Xiaoning Li

**Affiliations:** ^1^Department of Health Education, Jiangsu Provincial Center for Disease Control and Prevention, Nanjing 210009, China; ^2^School of Public Health, Nanchang University, Nanchang 330006, China; ^3^Jiangxi Province Key Laboratory of Preventive Medicine, Nanchang 330006, China; ^4^Department of Integrated Services, Jiangsu Provincial Center for Disease Control and Prevention, Nanjing 210009, China

## Abstract

The New Chinese Diabetes Risk Score (NCDRS) is one of the recommended tools for screening undiagnosed type 2 diabetes in China. However, its performance in detecting undiagnosed diabetes needs to be verified in different community populations. Also, it is unknown whether NCDRS can be used in detecting prediabetes. In the present study, we aimed to evaluate the performance of NCDRS in detecting undiagnosed diabetes and prediabetes among the community residents in eastern China. We applied NCDRS in 7675 community residents aged 18-65 years old in Jiangsu Province. The results showed that the participants with undiagnosed diabetes reported the highest NCDRS value, followed by those with prediabetes (*P* < 0.001). The best cut-off points of NCDRS for detecting undiagnosed diabetes and prediabetes were 27 (with a sensitivity of 78.0% and a specificity of 57.7%) and 27 (with a sensitivity of 66.0% and a specificity of 62.9%). The AUCs of NCDRS for identifying undiagnosed diabetes and prediabetes were 0.749 (95% CI: 0.739~0.759) and 0.694 (95% CI: 0.683~0.705). These results demonstrate the excellent performance of NCDRS in screening undiagnosed diabetes in the community population in eastern China and further provide evidence for using NCDRS in detecting prediabetes.

## 1. Introduction

With an increasing incidence and economic burden [1, 2], type 2 diabetes mellitus (DM) has been a significant public health concern worldwide. The global prevalence (age standardized) of DM has risen to 8.5% in the adult population in 2014 [3] and has grown fast in low- and middle-income countries over the past decade. Unfortunately, China is one of the countries facing serious challenges, with a prevalence of DM of 10.4% (World Health Organization, WHO 1999 criteria) in the population aged 18 and above in 2013 [4]. However, only 36.5% of the patients in China were aware of their diabetes condition [4]. There is an urgent need to apply valid measures for the screening of DM.

The other major issue is the detection of prediabetes (preDM), including impaired glucose tolerance (IGT) and impaired fasting glycemia (IFG). PreDM is a transition between normal glucose tolerance and diabetes, and it has been proved to be a critical high-risk factor of DM. The prevalence of preDM had reached 35.7% (American Diabetic Association, ADA 2003 criteria) in China in 2013 [4], which means that there may be a large number of patients with DM in the next few decades. However, the same as above, most of the individuals did not realize that they were at high risk of developing DM [5].

The previous studies demonstrate the significant roles of lifestyle intervention and early treatment in the prevention of DM [6–11], which highlight the importance of detecting DM and preDM at an early stage. However, the oral glucose tolerance test (OGTT) is complicated to implement, time-consuming, costly, and difficult to accept by most residents. In that, researchers have developed several DM risk assessments through nonlaboratory indicators for the rapid screening of undiagnosed DM in different populations [12–21]. There were also several DM risk measures developed for the Chinese population [22–25]. However, these measures were developed based on local conditions, and most of them did not consider issues such as gender differences. To provide a unified DM risk measurement, researchers developed a new assessment in 2013, the New Chinese Diabetes Risk Score (NCDRS) [26]. NCDRS is a DM risk assessment that includes age (years), gender, waist circumference (WC: cm), body mass index (BMI: kg/m^2^), systolic blood pressure (SBP: mmHg), and family history of DM [26]. It has been recommended as a unified tool for screening of undiagnosed DM in China [27]. However, two questions remain about the performance of NCDRS. First, only a few studies have reported on the efficacy of NCDRS in the detection of DM. Of these studies, most have suffered from small sample sizes and some were even conducted in a single clinic; others focused only on older adults [28, 29]. As we know, China is a multiethnic country with different economic levels and living habits. Research on the applicability of NCDRS in screening DM in different regions of China would be necessary. Second, to the best of our knowledge, no studies have evaluated the performance of NCDRS in screening preDM in a large sample of community residents in China. The efficacy of NCDRS in the detection of preDM requires further research.

Our study is aimed at filling the gap by evaluating the efficacy of NCDRS in the detection of undiagnosed DM and preDM among community residents in eastern China. In the present study, we conducted a community-based cross-sectional investigation in Jiangsu Province, eastern China. Urban and rural residents aged between 18 and 65 years old were investigated. This study will enhance our understanding of the performance of NCDRS in screening undiagnosed DM in the Chinese population and shed new light on its efficacy in detecting preDM.

## 2. Materials and Methods

### 2.1. Study Design and Population

A community-based cross-sectional study was conducted in Jiangsu Province of China from March to June 2016. The study population and procedures have been reported previously [30]. In brief, a four-stage stratified sampling method was used to select a representative sample of the general population in Jiangsu. A total of 8119 urban and rural residents aged between 18 and 65 years old were randomly selected from six districts or counties for the questionnaire survey, anthropometric measurement, and 2-hour oral glucose tolerance test (2 h-OGTT). People who have been diagnosed with diabetes, pregnancy, and severe mental disorders were excluded. Residents who failed to complete the questionnaire survey, anthropometric measurement, or blood specimen collection were excluded from data analysis. Finally, a total of 7675 residents were included in the analysis.

### 2.2. New Chinese Diabetes Risk Score (NCDRS)

The scores of NCDRS are shown in Table 1. The total score ranged from 0 to 51, and the recommended optimal cut-off point for undiagnosed DM was 25 [26].

### 2.3. Data Collection

The study was conducted in local township health centers by trained research staff. A standard questionnaire was used, including personal information such as sociodemographic characteristics and family history of DM. Anthropometric measurements were taken by trained observers, included body weight, height, waist circumference, and blood pressure.

### 2.4. Laboratory Measurements

A 5 mL blood specimen was obtained for fasting plasma glucose (FPG) measurement after at least 8 hours of fasting. The other 5 mL blood specimen was obtained for 2-hour plasma glucose (2 h PG) measurement after a 75 g anhydrous glucose load for two hours. As the six districts or counties were geographically distant, the laboratory tests of plasma glucose (PG) were carried out by local people's hospitals at the county or the center for disease control and prevention. The instruments and methods used to measure PG have been reported previously [30].

### 2.5. DM and PreDM Definition

According to the 1999 World Health Organization (WHO) criteria, DM was defined as FPG ≥ 7.0 mmol/L or 2 h PG ≥ 11.1 mmol/L. PreDM was defined as 6.1 mmol/L ≤ FPG < 7.0 mmol/L or 7.8 mmol/L ≤ 2 h PG < 11.1 mmol/L. Normal glucose tolerance was defined as FPG < 6.1 mmol/L and 2 h PG < 7.8 mmol/L.

### 2.6. Ethical Consideration

This study was approved by the Ethics Review Committee of Jiangsu Provincial Center for Disease Control and Prevention (No. JSJK2016-B003-03). Informed consent was obtained from each participant.

### 2.7. Statistical Analysis

Continuous and categorical variables were described by mean ± standard deviation (*X* ± SD) and percentage (%). According to different glucose metabolism levels, the characteristics of each group were described. Comparisons among the groups were made with one-way ANOVA for continuous variables and Chi-square test for proportions. Sensitivity, specificity, Youden index, positive predictive value, and negative predictive value were calculated for each NCDRS value from 25 to 32 for screening undiagnosed DM, preDM, or both of them. The optimal cut-off point was determined by the highest Youden index. The receiver operating characteristic (ROC) curves by gender and age groups were used to present the performance of NCDRS in screening both undiagnosed DM and preDM. The area under the curve (AUC) was used to evaluate the screening effect of NCDRS. All statistical analyses were performed in SPSS 17.0 and MedCalc18.11.3.

## 3. Results

The characteristics of the participants were presented in Table 2. The average age of the participants was 43.8 ± 11.9 years old, and 56.0% were female. Of the participants, 41.7% had a middle school education, and 39.0% were farmers. 16.8% of the participants reported that they had a family history of diabetes. Among all participants, the average BMI, WC, SBP, DBP, FPG, and 2 h PG were 25.1 ± 4.0 kg/m^2^, 83.5 ± 10.4 cm, 128.2 ± 18.8 mmHg, 79.2 ± 11.7 mmHg, 5.5 ± 1.0 mmol/L, and 6.6 ± 2.5 mmol/L, respectively. The average NCDRS score was 24.1 ± 9.3, and 52.1% of the participants reported a high-risk score (NCDRS ≥ 25). The detection rates of undiagnosed DM and preDM were 6.5% (95% CI: 5.96% to 7.07%) and 16.7% (95% CI: 15.91% to 17.58%). The detection rates for DM and preDM in the high-risk group (NCDRS ≥ 25) were 10.6% and 23.6%, while the detection rates in the low-risk group (NCDRS < 25) were 2.1% and 9.3%. By using the LSD method, the NCDRS scores of DM were higher than the scores of preDM (*P* < 0.001), and the scores of preDM were higher than the scores of normal glucose tolerance residents (*P* < 0.001). There was a certain relationship between the NCDRS scores and the glucose metabolism levels.

As shown in Table 3, the optimal cut-off point for the detection of undiagnosed DM was 31 for men (sensitivity = 65.2%, specificity = 69.0%) and 27 for women (sensitivity = 76.2%, specificity = 63.5%). For preDM, the optimal cut-off point was 29 for men (sensitivity = 61.1%, specificity = 64.7%) and 26 for women (sensitivity = 65.7%, specificity = 64.5%). When screening both undiagnosed DM and preDM, the best cut-off point was 27 for men (sensitivity = 73.5%, specificity = 55.6%) and 26 for women (sensitivity = 69.3%, specificity = 64.5%). In the total sample, the optimal cut-off point for screening undiagnosed DM was 27 (sensitivity = 78.0%, specificity = 57.7%, Youden index = 0.357), same as for the detection of preDM (sensitivity = 66.0%, specificity = 62.9%, Youden index = 0.289). At the cut-off point of 25 recommended by NCDRS, sensitivity, specificity, and Youden index of detecting undiagnosed DM were 84.8%, 50.1%, and 0.349, respectively. The PPV of NCDRS was 11.4% for DM and 20.8% for preDM.

**Figure 1:**
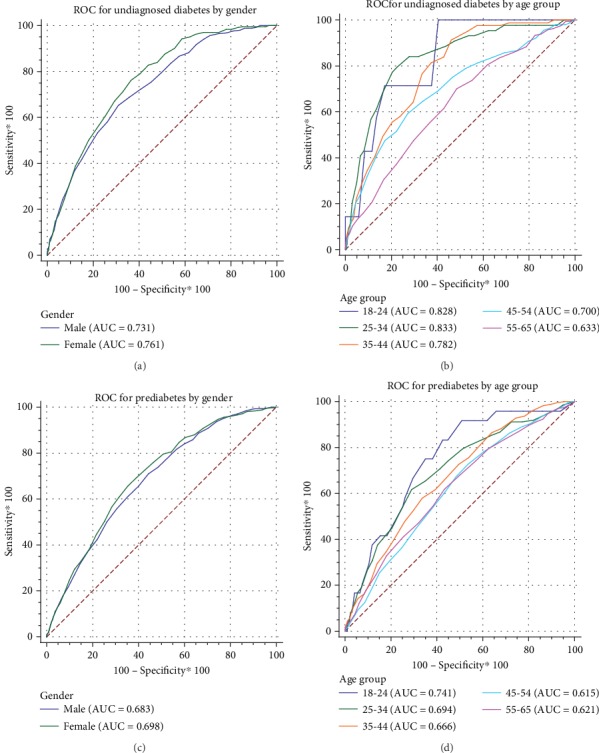
Receiver operating characteristic (ROC) curve for identifying undiagnosed diabetes (a and b) and prediabetes (c and d) by gender and age group in the study.

The areas under the ROC curves (AUCs) for identifying undiagnosed DM were 0.731 in men and 0.761 in women (*P* = 0.139). The AUC of the ≥45 age group was smaller than that of the other age groups (all *P* < 0.05) (see Figures 1(a) and 1(b)). The AUCs for identifying preDM were 0.683 in men and 0.698 in women (*P* = 0.325). There was a statistically significant difference in the AUCs between the 45-54 age group and the ≤44 age group (all *P* < 0.05) (see Figures 1(c) and 1(d)). The AUCs for identifying undiagnosed DM and preDM were 0.749 (95% CI: 0.739~0.759) and 0.694 (95% CI: 0.683~0.705). The AUC for identifying both undiagnosed DM and preDM was 0.718 (95% CI: 0.708~0.728) in the total sample.

## 4. Discussion

One of the aims of our study was to evaluate the performance of NCDRS in screening undiagnosed DM among community residents in eastern China. As we expected, NCDRS showed excellent validity in detecting undiagnosed DM. The AUC for identifying undiagnosed DM was 0.749 in this study, which was similar to 0.748 reported in the China National Diabetes and Metabolic Disorders Study [26]. However, the optimal cut-off point for undiagnosed DM in this study was 27, two points higher than the recommended cut-off point of 25 in the national survey above. In previous studies [28, 29], the best cut-off point was 33 in the population aged over 60 years and 20 in the hospital physical examination group. The possible cause for these differences may be attributed to the differences in age, regions, and other demographic characteristics among these findings. It indicates that, as we mentioned above, studies on the applicability of NCDRS in screening DM in different regions and populations of China is necessary. We found that, when using for the screening of undiagnosed DM, the sensitivity and specificity of NCDRS at the optimal cut-off point of 27 in this study were 78.0% and 57.7%; while at the recommended cut-off point of 25, the sensitivity and specificity were 84.8% and 50.1%. It demonstrates that, when using the cut-off value of 27 in community residents in eastern China, the sensitivity may decrease. Still, the specificity will improve, which could reduce the proportion of individuals who are misjudged as DM. Taken together, these findings suggest that a cut-off score of 27 is more applicable if we seek to maximize the effects of screening. However, given the severe consequences of missed diagnosis, a cut-off point of 25, so as to improve the sensitivity of detection, is also recommended in practice.

Moreover, NCDRS had a low PPV (11.4%) for DM in our study, which was similar to 11.8% reported in the national survey above [26]. As we know, more benefits of further diagnosis for individuals who were screening positive could be obtained as PPV increased. However, the PPV was closely related to the prevalence of the target disease in the tested population. When sensitivity and specificity were constant, PPV increased with the risen prevalence of the target disease. One previous study reported that the sensitivity and specificity of NCDRS were 70.5% and 53.0% in Chinese adults aged 45-70 with an undiagnosed DM prevalence of 15.3% [25], resulting in a PPV of 21.3%. It indicates that using NCDRS in a population with a higher prevalence of undiagnosed DM will result in higher benefits. Therefore, it is better to use NCDRS for screening the high-risk group of DM in the future.

To the best of our knowledge, this study is the first to evaluate the efficacy of NCDRS in screening preDM in a large sample of representative community residents in China. We found that the AUC of NCDRS in screening preDM was 0.694, indicating that NCDRS also performs well in the detection of preDM. Compared with the cut-off points for the detection of undiagnosed DM, the optimal cut-off scores for the detection of preDM were two points lower in men and one point lower in women. However, in the total sample, the optimal cut-off point for screening preDM was 27, which shared the same value with screening undiagnosed DM. It is an interesting finding that NCDRS may differ from other DM risk assessments. For example, in both the ADA scores and CDC scores, the cut-off points for preDM were one point lower than the points for DM [31]. It may be due to the fact that preDM shares common risk factors with DM in eastern China [32, 33]. Nevertheless, using the same cut-off point for the detection of DM and preDM makes NCDRS more convenient and applicable for practice.

There was no statistically significant difference in the AUC of NCDRS in detecting undiagnosed DM and preDM between men and women. However, the AUC showed statistically significant differences in identifying undiagnosed DM and preDM among different age groups. The performance of NCDRS was more effective in the ≤44 age group compared with the ≥45 age group in both DM and preDM. The previous study has shown that the DM risk assessments could neither provide efficient screening of DM, nor could they identify individuals with elevated blood glucose levels in an older population (≥55 years old) [34]. That is, like other assessments, NCDRS may be more applicable in a younger population. It means that NCDRS could play a significant role in early detection, early diagnosis, and early treatment, in which a higher return would be expected.

Several limitations to the current study need to be acknowledged. First, the tests of plasma glucose were conducted in different laboratories, which may result in slight differences in the value of plasma glucose. However, all of the tests were carried out by the local public hospital or the CDC. All laboratories of these institutes meet the requirements of the Administrative Measures for Clinical Laboratory of Medical Institutions. The instruments and reagents were also calibrated. Second, although we have taken efforts to collect samples from both urban and rural areas, the samples were only from one province in eastern China, which raises concerns about the possible selection bias. Further research in different regions is needed. Third, due to the nature of the cross-sectional study, we were unable to evaluate the performance of NCDRS in predicting DM and preDM events. We will try to conduct a follow-up study and assess the predictive validity of NCDRS further.

## 5. Conclusions

In conclusion, this study is the first to assess the efficacy of NCDRS in the screening of undiagnosed DM and preDM in a large sample of community residents in eastern China. The findings suggest that NCDRS performs well in screening both undiagnosed DM and preDM. Our study not only supports the excellent efficacy of NCDRS in the detection of undiagnosed DM but also highlights its potential usefulness in detecting preDM.

## Figures and Tables

**Table 1:** The scores of NCDRS.

Indicators	Score	Indicators	Score
Age (years)^a^		Waist circumference (cm)	
20-24	0	<75 (men) or <70 (women)	0
25-34	4	75-79.9 (men) or 70-74.9 (women)	3
35-39	8	80-84.9 (men) or 75-79.9 (women)	5
40-44	11	85-89.9 (men) or 80-84.9 (women)	7
45-49	12	90-94.9 (men) or 85-89.9 (women)	8
50-54	13	≥95 (men) or ≥90 (women)	10
55-59	15	Systolic blood pressure (mmHg)	
60-64	16	<110	0
65-84	18	110-119	1
BMI (kg/m^2^)		120-129	3
<22	0	130-139	6
22-23.9	1	140-149	7
24-29.9	3	150-159	8
≥30	5	≥160	10
Sex		Family history of diabetes, yes vs. no	
Women	0	No	0
Men	2	Yes	6

Note: ^a^participants aged 18 and 19 scored 0 for “Age” in the present study.

**Table 2:** Characteristics of the participants included in the study, by glucose metabolism levels.

	Overall (*n* = 7675)	Normal glucose tolerance (*n* = 5890)	PreDM (*n* = 1285)	DM (*n* = 500)	*P* value
Gender					
Male	3374 (44.0)	2486 (42.2)	632 (49.2)	256 (51.2)	<0.001^b^
Female	4301 (56.0)	3404 (57.8)	653 (50.8)	244 (48.8)	
Age (years)	43.8 ± 11.9	42.3 ± 11.9	48.3 ± 10.6	49.4 ± 10.0	<0.001^a^
Age group					
18-24	385 (5.0)	354 (6.0)	24 (1.9)	7 (1.4)	<0.001^b^
25-34	1620 (21.1)	1440 (24.4)	136 (10.6)	44 (8.8)	
35-44	1603 (20.9)	1284 (21.8)	238 (18.5)	81 (16.2)	
45-54	2552 (33.3)	1844 (31.3)	504 (39.2)	204 (40.8)	
55-65	1515 (19.7)	968 (16.4)	383 (29.8)	164 (32.8)	
Education level					
Below primary school	784 (10.2)	520 (8.8)	206 (16.0)	58 (11.6)	<0.001^b^
Primary school	1332 (17.4)	951 (16.1)	280 (21.8)	101 (20.2)	
Middle school	3203 (41.7)	2462 (41.8)	509 (39.6)	232 (46.4)	
High school	1630 (21.2)	1323 (22.5)	218 (17.0)	89 (17.8)	
College and above	726 (9.5)	634 (10.8)	72 (5.6)	20 (4.0)	
Occupation					
Public institution officer^c^	1061 (13.8)	854 (14.5)	162 (12.6)	45 (9.0)	<0.001^b^
Company employee	2231 (29.1)	1801 (30.6)	308 (24.0)	122 (24.4)	
Farmer	2995 (39.0)	2093 (35.5)	642 (50.0)	260 (52.0)	
Other	1388 (18.1)	1142 (19.4)	173 (13.5)	73 (14.6)	
Family history of diabetes					
Yes	1290 (16.8)	920 (15.6)	248 (19.3)	122 (24.4)	<0.001^b^
No	6385 (83.2)	4970 (84.4)	1037 (80.7)	378 (75.6)	
BMI (kg/m^2^)	25.1 ± 4.0	24.7 ± 3.9	26.3 ± 3.6	27.4 ± 3.9	<0.001^a^
WC (cm)	83.5 ± 10.4	82.2 ± 10.3	86.8 ± 9.7	90.3 ± 10.0	<0.001^a^
SBP (mmHg)	128.2 ± 18.8	125.7 ± 18.0	134.9 ± 18.9	140.4 ± 18.1	<0.001^a^
DBP (mmHg)	79.2 ± 11.7	77.8 ± 11.4	82.8 ± 11.7	85.2 ± 10.9	<0.001^a^
FPG (mmol/L)	5.5 ± 1.0	5.2 ± 0.4	5.9 ± 0.6	7.8 ± 2.1	<0.001^a^
2 h PG (mmol/L)	6.6 ± 2.5	5.6 ± 1.0	8.2 ± 1.5	13.4 ± 4.1	<0.001^a^
NCDRS value	24.1 ± 9.3	22.5 ± 9.2	28.7 ± 7.4	31.5 ± 6.7	<0.001^a^
NCDRS group					
Low-risk (NCDRS < 25)	3673 (47.9)	3255 (55.3)	342 (26.6)	76 (15.2)	<0.001^b^
High-risk (NCDRS ≥ 25)	4002 (52.1)	2635 (44.7)	943 (73.4)	424 (84.8)	

Note: data are reported as mean ± standard deviation or frequency (percent (%)); BMI=body mass index; WC=waist circumference; SBP=systolic blood pressure; DBP=diastolic blood pressure; FPG=fasting plasma glucose; 2 h PG=2 hour plasma glucose. ^a^The *P* value of one-way ANOVA. ^b^The *P* value of the Chi-square test. ^c^Public institution officers include government officers, teachers, medical staffs, and other public institution officers.

**Table 3:** Sensitivity, specificity, Youden index, positive predictive value (PPV), and negative predictive value (NPV) of NCDRS cut-off points in identifying undiagnosed DM and preDM in men and women in the study.

Cut-off point	Men					Women					Total				
	Sensitivity%	Specificity%	Youden index	PPV%	NPV%	Sensitivity%	Specificity%	Youden index	PPV %	NPV %	Sensitivity%	Specificity%	Youden index	PPV %	NPV %
DM^a^															
25	86.7	42.6	0.293	11.0	97.5	82.8	56.0	0.387	10.2	98.2	84.8	50.1	0.349	10.6	97.9
26	83.6	46.3	0.299	11.3	97.2	79.1	59.6	0.387	10.6	97.9	81.4	53.9	0.353	10.9	97.7
27	79.7	50.2	0.299	11.6	96.8	**76.2**	**63.5**	**0.397**	**11.2**	**97.8**	**78.0**	**57.7**	**0.357**	**11.4**	**97.4**
28	75.4	54.8	0.302	12.1	96.4	71.3	67.1	0.384	11.5	97.5	73.4	61.7	0.351	11.8	97.1
29	72.3	59.5	0.318	12.8	96.3	67.2	70.7	0.379	12.1	97.3	69.8	65.8	0.356	12.5	96.9
30	68.8	64.3	0.330	13.6	96.2	60.7	74.7	0.354	12.6	96.9	64.8	70.2	0.350	13.2	96.6
31	**65.2**	**69.0**	**0.342**	**14.7**	**96.0**	55.3	78.3	0.336	13.3	96.7	60.4	74.3	0.347	14.1	96.4
32	58.2	73.8	0.320	15.4	95.6	50.0	82.1	0.321	14.4	96.5	54.2	78.5	0.327	14.9	96.1
PreDM^b^															
25	77.5	47.7	0.252	27.4	89.3	69.4	60.8	0.301	25.4	91.2	73.4	55.3	0.286	26.4	90.5
26	73.9	51.5	0.254	27.9	88.6	**65.7**	**64.5**	**0.302**	**26.2**	**90.7**	69.7	59	0.287	27.1	89.9
27	71.0	55.6	0.267	28.9	88.3	61.1	68.2	0.293	26.9	90.1	**66.0**	**62.9**	**0.289**	**28.0**	**89.5**
28	65.7	60.0	0.257	29.5	87.3	56.5	71.6	0.281	27.6	89.6	61.0	66.7	0.277	28.6	88.7
29	**61.1**	**64.7**	**0.258**	**30.6**	**86.7**	50.4	74.7	0.251	27.7	88.7	55.6	70.5	0.262	29.2	87.9
30	55.9	69.4	0.252	31.7	86.1	44.6	78.4	0.230	28.4	88.1	50.1	74.6	0.247	30.1	87.3
31	50.0	73.8	0.238	32.7	85.3	38.1	81.5	0.196	28.3	87.3	44.0	78.2	0.222	30.6	86.5
32	42.6	77.9	0.205	32.9	84.2	33.1	85.0	0.180	29.7	86.9	37.7	82.0	0.197	31.4	85.8
PreDM/DM^c^															
25	80.2	47.7	0.278	35.4	87.1	73.0	60.8	0.338	32.9	89.5	76.6	55.3	0.318	34.2	88.6
26	76.7	51.5	0.282	36.1	86.1	**69.3**	**64.5**	**0.339**	**34.0**	**88.9**	73.0	59.0	0.320	35.1	87.8
27	**73.5**	**55.6**	**0.292**	**37.2**	**85.5**	65.2	68.2	0.334	35.1	88.2	**69.4**	**62.9**	**0.322**	**36.2**	**87.1**
28	68.5	60.0	0.285	38.0	84.2	60.5	71.6	0.321	36.0	87.3	64.5	66.7	0.312	37.0	86.1
29	64.3	64.7	0.290	39.4	83.5	55.0	74.7	0.297	36.4	86.3	59.6	70.5	0.301	38.0	85.2
30	59.6	69.4	0.290	41.0	82.8	48.9	78.4	0.273	37.4	85.4	54.2	74.6	0.288	39.3	84.3
31	54.4	73.8	0.282	42.6	81.9	42.8	81.5	0.243	37.8	84.4	48.6	78.2	0.268	40.3	83.4
32	47.1	77.9	0.250	43.2	80.5	37.7	85.0	0.226	39.8	83.8	42.4	82.0	0.244	41.6	82.4

Note: DM=diabetes mellitus; preDM=prediabetes. ^a^Detecting undiagnosed diabetes among all participants. ^b^Detecting prediabetes among participants excluding undiagnosed diabetes. ^c^Detecting undiagnosed diabetes and prediabetes among all participants.

## Data Availability

The data used to support the findings of this study are available from the corresponding author upon request.
